# Frequent Heterogeneous Missense Mutations of GGAP2 in Prostate Cancer: Implications for Tumor Biology, Clonality and Mutation Analysis

**DOI:** 10.1371/journal.pone.0032708

**Published:** 2012-02-28

**Authors:** Yi Cai, Jianghua Wang, Chengxi Ren, Michael Ittmann

**Affiliations:** 1 Department of Pathology and Immunology, Baylor College of Medicine, Houston, Texas, United States of America; 2 Michael E DeBakey Department of Veterans Affairs Medical Center, Houston, Texas, United States of America; National University of Ireland Galway, Ireland

## Abstract

Prostate cancer is the most common visceral malignancy in Western men and a major cause of cancer deaths. Increased activation of the AKT and NFkB pathways have been identified as critical steps in prostate cancer initiation and progression. GGAP2 (GTP-binding and GTPase activating protein 2) is a multidomain protein that contains an N-terminal Ras homology domain (GTPase), followed by a PH domain, a C-terminal GAP domain and an ankyrin repeat domain. GGAP2 can directly activate signaling via both the AKT and NFkB pathways and acts as a node of crosstalk between these pathways. Increased GGAP2 expression is present in three quarters of prostate cancers. Mutations of GGAP2 have been reported in cell lines from other malignancies. We therefore analyzed 84 prostate cancer tissues and 43 benign prostate tissues for somatic mutations in GGAP2 by direct sequencing of individual clones derived from the GAP and GTPase domains of normal and tumor tissue. Overall, half of cancers contained mutant GAP domain clones and in 20% of cancers, 30% or more of clones were mutant in the GAP domain. Surprisingly, the mutations were heterogeneous and nonclonal, with multiple different mutations being present in many tumors. Similar findings were observed in the analysis of the GTPase domain. Mutant GGAP2 proteins had significantly higher transcriptional activity using AP-1 responsive reporter constructs when compared to wild-type protein. Furthermore, the presence of these mutations was associated with aggressive clinical behavior. The presence of high frequency nonclonal mutations of a single gene is novel and represents a new mode of genetic alteration that can promote tumor progression. Analysis of mutations in cancer has been used to predict outcome and guide therapeutic target identification but such analysis has focused on clonal mutations. Our studies indicate that in some cases high frequency nonclonal mutations may need to be assessed as well.

## Introduction

A variety of genetic and epigenetic alterations have been described in prostate cancer. Numerous studies have found consistent patterns of copy number alterations such as loss of 8p and 13q14 and gain of 8q24 in clinically localized and advanced prostate cancers [1], [2]. Epigenetic alterations such as methylation are also common in prostate cancer. In contrast, most studies to date have shown only infrequent clonal point mutations in clinically localized prostate cancer [2], [3]. In more advanced prostate cancers, clonal point mutations of tumor suppressor genes such as PTEN [4] and p53 [3] are more common, in contrast to the low frequency of mutation of these genes in localized cancer [5], [6], but are still not common compared to most malignancies. Activating clonal mutations in oncogenes, such as RAS, are not common in prostate cancer in the US [3], in contrast to the more frequent mutation observed in other common human cancers such as colon and lung cancer. Clonal androgen receptor mutations are seen in castrate resistant prostate cancer and appear to be selected for as a mechanism by which prostate cancer cells can survive in low androgen environment [3]. Thus available data indicate that clonal point mutations, particularly of oncogenes, are rare in clinically localized prostate cancer.

GGAP2 (also known as PIKE-A) is a G-protein which has a strong GTPase activity, as expected from its RAS homology domain. It also contains a GAP domain can activate the GTPase activity via either intramolecular or intermolecular interaction. GGAP2 binds to activated AKT and strongly enhances its activity and this interaction is promoted by GTP binding [7]. We have shown that activated AKT can bind and phosphorylate GGAP2 at serine 629, which enhances GTP binding by GGAP2 and AKT activation [8]. Phosphorylated GGAP2 can also bind the p50 subunit of NFkB and enhances NFkB transcriptional activity. Increased activation of the phosphatidyl-inositol-3 kinase/AKT and NFkB pathways have both been identified as critical pathways in cancer initiation and progression in a variety of human malignancies, including prostate cancer. We have demonstrated significantly increased expression GGAP2 in the majority of human prostate cancers [8]. When GGAP2 is expressed in prostate cancer cells it enhances proliferation, focus formation in vitro and tumor progression in vivo. Thus increased GGAP2 expression, which is present in three quarters of human prostate cancers, can activate two critical pathways that have been linked to prostate cancer initiation and progression and can enhance tumor progression in vivo.

Hu et al have identified mutant forms of GGAP2 in sarcoma, neuroblastoma and glioblastoma cell lines [9]. In vitro studies show these mutant forms have enhanced GTPase activity and more strongly activate AKT than wild-type GGAP2. Consistent with these observations the GGAP2 mutants promote growth of glioblastoma cells and transformation of NIH3T3 cells [10]. We therefore sought to identify mutations of GGAP2 in human prostate cancer samples. We have found high frequencies of missense GGAP2 mutations in clinically localized human prostate cancer. Surprisingly, the mutations are heterogeneous and nonclonal, with multiple different mutations being present in many tumors. The presence of these mutations was associated with aggressive clinical behavior and increased AP-1 transcriptional activity. Thus, GGAP2 is the most commonly mutated oncogene in human prostate cancer to date but the mutations are heterogeneous rather than clonal, implying marked clonal heterogeneity in clinically localized human prostate cancers. The presence of high frequency nonclonal mutations of a single gene is novel and represents a new mode of genetic alteration that can promote tumor progression.

## Results

### Mutation analysis of the GAP domain of GGAP2

To determine if GGAP2 is mutated in prostate cancer we initially focused on the GAP domain, since this region is an important negative regulator of GGAP2 activity. We analyzed cDNAs from 15 cancers and 9 benign prostate tissues from radical prostatectomy specimens. The GAP domain was amplified and individual clones were isolated and sequenced. Results are shown in Table 1. Twelve of fifteen cancer cases had at least one clone with a GGAP2 missense and/or stop mutations while only 2 of 9 benign cases had such mutations. The benign cases had only a single mutant clone each while up to 42 percent of clones in the cancer cases were mutated. Overall 38 of 206 clones from the cancer tissues were mutant versus 2 of 137 in benign. This difference was highly statistically significant (p<0.001, chi square). To rule out an artifact due to reverse transcription or the possibility that mutant transcripts may be transcribed preferentially or have increased stability we directly analyzed the GAP domain in genomic DNAs from 46 cancers and 22 benign tissues. As shown in Table 1, 20 of 46 cancer tissues contained at least one mutant clone and in 12 of 46 cancer tissues more than 30% of clones contained missense or stop mutations. Only a single mutant clone was identified from the benign tissues. Overall, 52 of 334 clones from the cancer tissues were mutant versus 1 of 167 from the benign tissues (p<0.001, chi square). Combining cDNA and genomic analysis, 32 of 61 cancer cases contained clones with GAP domain mutations and in 14 cases 30% or more of the clones were mutant.

**Table 1:** Mutation analysis of the GAP domain of GGAP2 in prostate cancer.

cDNA							Missense		Stop
ID	Type	Clones	Mutations	Percent					
7357	Cancer	19	8	42	E656G	V711M	I589F	S584A	
					L624P	E696G	L630P		W703X
1954		17	5	29	L624M	E672G	L643P	S705T	
					AS692G				
11686		20	4	20	S666P/K681E	N648S	G594R/L632W		
10420		12	4	33	R653C	A705G	R679H	V711M	
11147		14	3	21	D649G	I678T	T640A		
9523		18	3	17	G621D	G594E	A580V/A607T		
6882		14	3	21	L641P	A645D			Q709X
6098		7	2	29	D710V	L702P			
6511		10	2	20	R662C	T622I			
3689		12	2	17	T599P	L686P			
8032		13	1	8	C593R				
12375		13	1	8	L698P				
97	Benign	16	1	6	S670P				
11627		16	1	6	L620V				
**Genomic**									
19334	Cancer	8	5	63	E638G	W600R	I611V	E612K	
					A721V				
27312		8	4	50	A663P	Q707R	I581V	V576A	
11537		9	4	44	D633G	Q684R	S625P	A651G	
23536		10	4	40	N648Y	V591L	G586R	Q707R	
47974		9	4	44	E612V	V642G	I646L	L639M	
17557		6	3	50	K664Q	I609T	G621C		
6337		7	3	43	T659A	I609T	A651G		
20088		7	3	43	S657G	V706M	I611V		
17125		8	3	38	I581V	R582P	I581V		
18099		10	3	30	K664Q	H623R	A607V/E696G		
22766		10	3	30	E612Q	G621S	S602N		
18062		10	3	30	S692G	T569A	A544T		
10702		7	2	29	Y682C	Q707R			
21918		8	2	25	A708V	A708V			
8665		10	1	10	W600L/S629L				
27804		8	1	12	N652I/L686P				
29823		8	1	12	R653C				
26065		6	1	17	S629P				
24069		10	1	10	D592G				
29886		10	1	10					Q707X
25909	Benign	9	1	11	T659A				

Missense and stop mutations cancer and benign tissues are shown using the format: normal amino acid/amino acid number in GGAP2/mutant amino acid. An X indicates a stop mutation. Only tissues with missense or stop mutations are shown.

Surprisingly we found that the missense mutations were highly heterogeneous. There were no recurrent missense mutations involving more than two tumors. In tissues with multiple mutant clones there were only two cases with two identical mutant clones. There was variability in the distribution of the missense mutations with the regions between amino acids 640–660 and 700–710 having relatively more frequent mutations while mutations were uncommon from amino acids 540–570 but there was no statistically significant “hot spots”. In several clones we found 2 mutations in the same clone. This is similar to the observation of Hu et al [9], who found multiple mutations in several mutant GGAP2 cDNAs isolated from sarcoma and glioblastoma cell lines. In addition to the multiple missense mutations, we observed 3 stop mutations, all at the carboxy terminal portion of the GAP domain (aa 703–709) which is located toward the carboxy terminus of the GGAP2 protein and would result in a truncated protein. Of note, Hu et al [9] found a truncation at amino acid 756 in the GGAP2 cDNA from CRL-2098 osteosarcoma cells.

Given this surprising heterogeneity we considered the possibility that this may represent a PCR misincorporation artifact. However, we found only 3 silent mutations among 540 clones from cancer tissues (versus 90 missense or stop) while in the benign tissues we found 2 silent mutations among 304 clones (versus 3 missense mutations). The proportion of missense and stop versus silent mutation was much higher in the cancer tissue than in the benign tissue and the difference was statistically significant (p = 0.02, Fisher exact test). This is inconsistent with a random misincorporation. To further examine this point, we systematically determined the consequences of transition mutations on amino acid sequence for all nucleotides in the GAP domain. We only examined transitions since 82% of the observed mutations were transition mutations (data not shown). Systematic transition mutation of each nucleotide in the GAP domain would yield 273 missense, 15 stop mutations and 162 silent mutations. The difference in the proportions of missense and stop versus silent mutations we observed (90 and 3) compared the predicted distribution (288 and 167) was highly statistically significant (p<0.001, chi sq). Finally, we considered the possibility that the cancer tissues had an increase rate of mutation targeting the first and second bases of each codon resulting in random missense mutation in all genes. We therefore examined 5 cancer and 5 benign tissues for mutations in β-actin. We found no mutations in 32 clones from cancer tissue and 36 clones from benign tissues. The proportion of missense and stop clones in GGAP2 was statistically significantly higher than in β-actin (p = 0.02, chi sq). Thus the observed heterogeneous mutations in the GAP domain of GGAP2 are indeed genuine.

### Mutation analysis of the GTPase domain of GGAP2

The GTPase domain is also a key regulator of GGAP2 activity. We therefore examined cDNAs from 23 cancers and 12 benign tissues for GTPase domain mutations. The results were very similar to those observed with GAP domain (Table 2). Fifteen of 23 cancers contained missense mutations versus 1 of 12 benign tissues. In four cancer cases, 40% or more of clones were mutant, while only a single mutant clone was observed in the benign tissue. Overall, 28 of 188 clones from the cancer tissues were mutated versus 1 of 88 in benign tissue (p<0.001, chi sq). The overall pattern of mutations in the cancer tissue was similar to the GAP domain in that mutations were highly heterogeneous, both within a single cancer tissue and between cancer tissues. We found one double mutant clone, similar to the GAP domain. Of note, no stop mutations were observed. Given the amino terminal location of the GTPase domain, any stop mutations would almost certainly yield inactive protein since it would lack the PH domain. We found only 2 silent mutations, one in a cancer and one from benign tissue. In nine tissues of 35 analyzed (26%) we detected multiple clones containing a previously described silent polymorphism (Rs17852479) at L246 which does not result in any amino acid change. This polymorphism occurs in approximately 28% of individuals in previously studied populations, similar to our finding. A summary of the mutation analysis of GGAP2 is shown in Table 3.

**Table 2:** Mutation analysis of the GTPase domain of GGAP2 in prostate cancer.

ID	Tissue	Clones	Mutations	Percent		Missense	mutations	
10764	Cancer	9	4	44	V364A	L239P	P300S	R182G
6346		10	4	40	E281G	S275P	A198V	S302P
12161		10	4	40	A173T	N265D	E167K	H117R
4343		5	2	40	Q115R	L246F		
8032		9	2	22	H268R/L234P	Q262R		
7357		10	2	20	R234C	S329N		
8748		8	2	25	S207N	C200G		
1954		8	1	12	E332G			
3230		7	1	14	K360E			
3689		8	1	12	R310G			
6882		10	1	10	A292V			
9560		7	1	14	S329G			
11147		6	1	17	F223L			
14198		7	1	14	A175T			
15250		7	1	14	K229E			
1610	Benign	12	1	8	H117R			

The GTPase domain was cloned from cDNAs from prostate cancer (>70% cancer) or benign peripheral zone tissues and sequenced. A total of 23 cancers and 12 benign tissue samples were analyzed. The number of clones is indicated as is the number and percentage of clones with missense mutations. For each individual tissue the missense mutations are shown using the format: normal amino acid/amino acid number in GGAP2/mutant amino acid. Only tissues with missense mutations are shown.

**Table 3:** Summary of mutation analysis of GAP and GTPase.

Tissue	DNA Analyzed	Number of Tissues	Total Clones	Missense/Stop	Silent
Cancer	GAP (cDNA)	15	206	38	2
	GAP (genomic)	46	334	52	1
	GTPase (cDNA)	23	188	28	1

	DNA Analyzed	Number of Tissues	Total Clones	Missense/Stop	Silent
Benign	GAP (cDNA)	9	137	2	2
	GAP (genomic)	22	167	1	0
	GTPase (cDNA)	12	88	1	1

Summary of mutation analysis of GAP and GTPase clones from cDNA or genomic DNAs from prostate cancer or benign prostate tissues. Does not include the known germline polymorphic loci.

### GAP Domain Mutations Increase AP-1 Transcriptional Activity

**Figure 1:**
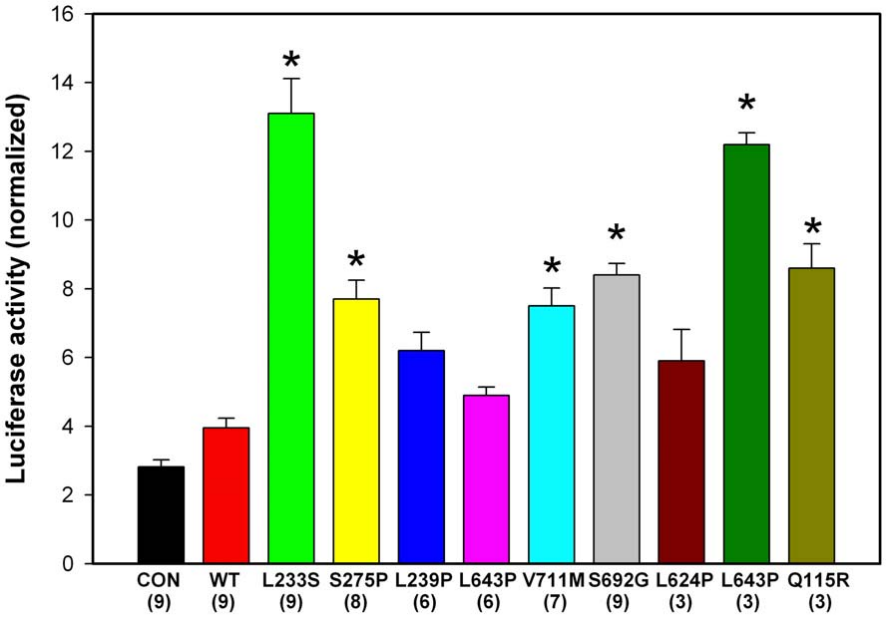
GGAP2 mutations result in enhanced transcription from AP-1 reporter constructs. Asterisks indicate statistically significant increase relative to wild-type (WT) GGAP2 by ANOVA (p<.05). Mean +/−SEM. Mutation and number of transfections are shown.

We have shown that NFκB can increase expression of FOS in prostate cancer cells and thus AP-1 activity [11]. To test whether GGAP2 and mutant GGAP2 impacted AP-1 transcription we used site directed mutagenesis to engineer GGAP2 expression constructs containing 9 different missense mutations. These mutant constructs or wild-type or empty vector controls were co-transfected with an AP-1 reporter construct into 293T cells and normalized luciferase activity measured. As shown in Figure 1, wild type GGAP2 modestly increases AP-1 driven transcription. Multiple mutant clones demonstrated marked enhancement of AP-1 promoter transcription in cells transfected with mutant when compared to wild-type GGAP2 (Fig. 1).

### Association of GAP domain mutations with clinical and pathological parameters of aggressive disease

**Figure 2:**
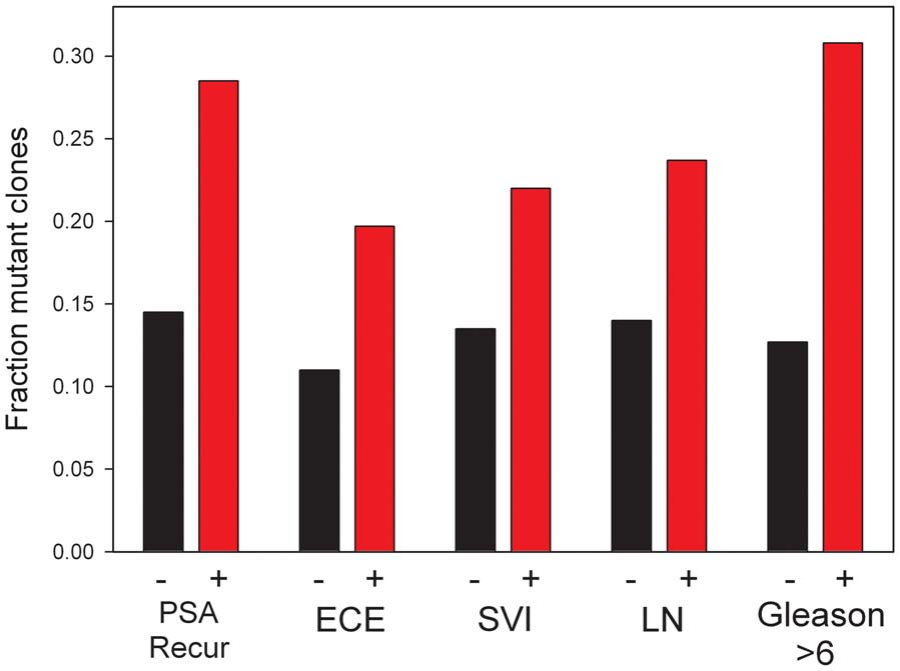
Association of GAP domain mutations with clinical and pathological parameters associated with aggressive prostate cancer. The fraction of clones containing missense or stop mutations for cases with each indicated clinical or pathological parameter is shown. All differences between pathological and clinical variables were statistically significant. Specifically: for early PSA recurrence (<2 years post surgery) versus no or late recurrence (p<0.001, chi sq); extracapsular extension (ECE) versus no ECE (p = 0.015, chi sq); seminal vesicle invasion (SVI) versus no SVI (p = 0.027, chi sq); pelvic lymph node metastasis (LN) versus no metastasis (p = 0.027, chi sq); Gleason 5/6 versus 7–10 (p = 0.002, chi sq).

To determine whether the presence of missense or stop mutations in the GAP domain were associated with aggressive disease we examined in proportion of such mutant clones in prostate cancers with various clinical and pathological parameters associated with aggressive disease (Figure 2). Early PSA recurrence after radical prostatectomy is associated with death from disease [12]. Cancers with early PSA recurrence had 49 mutations among 172 clones analyzed, while cases without early PSA recurrence had only 34 mutations in 234 clones. This difference was statistically significant (p<0.001, chi sq). Consistent with this, we also found significantly increased proportions of GAP mutations in cases with pelvic lymph node metastasis (p = 0.027, chi sq), seminal vesicle invasion (p = 0.027, chi sq), extracapsular extension (p = 0.015, chi sq) and higher Gleason score (Gleason 5/6 Versus 7–10, p = 0.002, chi sq). These findings strongly support the concept that GAP domain mutations in GGAP2 can promote prostate cancer progression.

## Discussion

Clonal mutations in clinically localized prostate cancer are uncommon and usually involve tumor suppressor genes (reviewed in [3]). Mutations in oncogenes such as RAS are uncommon in US men with prostate cancer although RAS mutations have been identified more commonly in prostate cancers from Japanese men [3]. We have identified frequent mutations of GGAP2 in localized prostate cancer. Overall, half of cancers contained at least one mutant GAP domain clone and in 20% of cancers, 30% or more of clones were mutant in the GAP domain. Surprisingly, while there were 10 different recurrent mutations these only recurred 2–3 times each, overall the GAP domain mutations were heterogeneous and nonclonal. Similar findings were observed in the analysis of the GTPase domain. Multiple lines of evidence argue that these finding are not an artifact including: the rarity of mutation in benign prostate tissues; the dominance of missense mutations in the cancer tissues; the paucity of silent mutations in cancer tissues and the absence of mutations in β-actin.

While both overexpression and nonclonal mutation of GGAP2 are common in prostate cancer the relationship between these two alterations is unclear. Both can potentially activate the siganaling activities of GGAP2 in prostate cancer, although detailed studies would be needed to discern whether these activities are the same for different specific mutations. In some cases overexpression might potentially enhance the biological activities associated with mutation although it is also possible that mutation may compensate for lack of overexpression. Detailed studies of GGAP2 expression, nonclonal mutation and markers of pathway activation in large numbers of tumors will be needed to understand the impact of these distinct alterations in prostate cancer.

Intratumoral genetic heterogeneity involving point mutations of genes such as p53 or K-RAS in different regions of single macroscopic tumors has been noted in cancers such as colon cancer [13] and gliomas [14]. It should be noted that in our cases all tumors represent a single 6 mm tumor focus and thus our cancers all were from a single tumor focus and is thus the heterogeneity we observed is distinct from this geographic genetic heterogeneity. In our case, the observed heterogeneity reflects heterogeneity at the cellular level within a single tumor focus.

Are the mutations we observed significant? The missense mutation frequency observed in the GAP domain in cancer tissues was 370×10^−6^ per bp sequenced and for the GTPase domain 298×10^−6^ per bp. Bielas et al [15] have shown that the frequency of random mutation in cancer tissues is approximately 2.1×10^−6^ per bp across multiple cancer types. Thus our observed frequency for missense mutation in GGAP2 is 100-fold higher than the background rate of mutation, strongly implying selective growth advantage for the mutant clones. We have also found a significant association between the frequency of mutation in the GAP domain and clinical and pathological parameters associated with aggressive disease, indicating they are clinically significant. It should be noted that in 20% of cases examined that more than 30% of clones from cancer were mutant in the GAP domain. Given that the tissues analyzed were approximately 80% cancer on average, at least 75% of cancer cells would contain a mutant allele (assuming one mutation per cell) in such cases. This is a minimum figure since it does not include GTPase domain mutations and potential mutations in other regions of GGAP2, which have been reported [9]. Thus the observed high frequency heterogeneous mutations could contribute directly to local tumor growth in many cases. In addition, the most potent mutations may promote metastasis of specific cellular clones. There is evidence to support the concept that nonclonal p53 mutations in primary prostate cancers can give rise to metastatic lesions [16]. The high frequency of diverse nonclonal mutations in GGAP2 may provide numerous potential metastatic clones.

Most studies of mutations in cancer have justifiably focused on clonal mutations since it is easier to see the significance of such mutations. Heterogeneous nonclonal mutations will not be detected by many analytical methods or are not further analyzed since it is unclear whether they may be PCR artifacts or simply passenger mutations. Our findings indicate that in some cases high frequency heterogeneous nonclonal mutations can occur and may be clinically important. It remains to be determined how often this is the case with other tumor suppressor genes and oncogenes. In some cases groups have analyzed primary prostate cancers for the presence of mutation using single stranded conformation polymorphism assays followed by sequencing of abnormally migrating bands and found relatively high rates of mutation in some genes. For example, using this approach, mutations in plexin-B1 in were identified in 46% of primary prostate cancers [17] but it is difficult to determine the exact percentage of tumor cells in a tumor with that mutation. Given that the mutations are frequent enough to give a distinct band on single stranded conformation polymorphism assays they must be quite frequent although not clonal. This is in contrast to our findings in GGAP2 in which the mutations are highly heterogeneous. Thus variable levels of nonclonal mutations, from highly heterogeneous to oligoclonal may exist in prostate cancer. On the other hand, using an approach similar to ours, Steinkamp et al [18] sequenced androgen receptor mRNAs from castrate resistant prostate cancer metastasis. They found high levels of heterogeneity in the mutations with many mutations being present in only 5–10% of clones. This finding is similar to what we observed in GGAP2. The androgen receptor plays a central role in prostate cancer pathogenesis and survival so there is strong selective pressure to retain mutations that lead to activity in the face of anti-androgen therapies. We have shown that GGAP2 is frequently overexpressed in prostate cancer and can activate two key pathways in prostate cancer progression i.e. the NFκB and AKT pathways. In addition, it has a relatively large negative regulatory domain that may be susceptible to disruption, which may make it far easier to activate than some oncogenes such as RAS that require specific point mutations. Additional analyses will be needed to determine the extent to which other genes, including tumor suppressor genes and other oncogenes, have high frequency non-clonal missense or stop mutations.

The potential for high frequency nonclonal mutation adds another layer of complexity to the complex mutational landscape of common cancers that has been revealed by large scale sequencing [19], [20], [21]. Of note, it has been shown that nonclonal mutations in K-RAS in lung cancer treated with tyrosine kinase inhibitors significantly impact survival [22]. Thus it will be important to determine the extent to which nonclonal mutations occur across of broad range of genes in prostate and other cancers and whether they impact survival and response to therapy.

## Materials and Methods

### Human tissue samples

Normal peripheral zone and cancer tissues were collected with written informed consent from men undergoing radical prostatectomy by the Baylor College of Medicine Prostate Cancer Program Tissue Bank and snap frozen as described previously [23]. Patients ranged in age from 43–73 years of age and were predominantly Caucasian. In all cases preoperative imaging and clinical examination revealed clinically localized disease. Pathological staging of radical prostatectomy specimens and pelvic lymph nodes showed approximately 30% Stage 2 (T2N0); 50% Stage 3 (T3N0) and 20% Stage 4 (Any T, N1). All patients provided written informed consent to donate tissues for research and these studies were approved by the Baylor College of Medicine Institutional Review Board. Benign tissues were confirmed to be free of cancer and cancer tissues contained at least 70% carcinoma. RNAs and DNAs were extracted as described previously [24], [25]. PSA recurrence was defined as serum PSA>0.2 ng/ml, with early recurrence being recurrence within 2 years of surgery.

### Mutation analysis

The N-terminal GTPase domain and the C-terminal GAP domain of GGAP2 gene were amplified using PCR and cloned into the PCR 2.1 TOPO vector using TOPO TA cloning kit ( Invitrogen). PCR was performed using Platinum Taq (Invitrogen) to minimize misincorporation. Primers used for cloning were: for GTPase domain Forward: CCGCTCCATTCCTGAACTG; Reverse: GTTGCTGCTTGCGCAAG for the GAP domain: Forward: CACAGACAGCCAAAGCGA; Reverse: CCAAAAGCAGGAGAACGGTAG. DNAs were sequenced in both directions and all base pair changes called by the machine read of the sequence were confirmed by visual examination of sequencing traces. Clones with poor quality sequencing traces were not analyzed. No novel reportable germ line variants were detected.

### Site directed mutagenesis

Single nucleotide mutagenesis was carried out according to the manufacturer's protocol (Stratagene). Briefly, primers with the target mutations were used in PCR to generate GGAP2 expression constructs containing 9 different missense mutations. Primers used are shown in Table 4. Dpn1enzyme was added to PCR products for 1 h at 37°C to digest template plasmid DNA before the transformation. Clones were sequenced to verify the mutations.

**Table 4:** Primers for site directed mutagenesis of GGAP2.

L233S	For	GAAGGTGGTGACCTCGCGCAAGCAGCAACA
	Rev	TGTTGCTGCTTGCGCGAGGTCACCACCTTC
S275P	For	CGACTACTCTTCTCCCCTCCCGTCCTCACC
	Rev	GGTGAGGACGGGAGGGGAGAAGAGTAGTCG
L239P	For	GCCTCTGGCTGCCTGCAAGTCCCTGC
	Rev	CCAGAGGCTGTTGCTGCTTGCGCAAGG
L643P	For	GCCACGGGAGCTGACCCTGGTGCCGACGGC
	Rev	GCCGTCGGCACCAGGGTCAGCTCCCGTGGC
V711M	For	CATGGCTACCGTTCTCCTGC
	Rev	CATGTCCTGGGCCTGCAC
S692G	For	GGGCACCTCGGAGGAGC
	Rev	CCAGCGGCGCCAGGAA
L624P	For	CCGTCCCGCGTTCGCT
	Rev	CGGGTGTGTGCCCAGGTT
L643P	For	GCCACGGGAGCTGACCCTGGTGCCG
	Rev	GCCGTCGGCACCAGGGTCAGCTCCC
Q115R	For	GTTGGTGGATGGACGGACACATCTGGTGCT
	Rev	AGCACCAGATGTGTCCGTCCATCCACCAAC

### Luciferase transcriptional reporter assays

Luciferase transcriptional reporter assays were performed as described previously [11] using 293T cells. Both AP-1 luciferase reporter vector and pRL Renilla Luciferase vector were obtained from Stratagene (Cat# 219077 and #E2810). The pRL Renilla Luciferase Reporter Vectors are intended for use as an internal control reporters in combination with AP-1 to cotransfect 293T cells. Transient transfection was conducted in triplicate in 24-well plates. Luciferase activity was determined and normalized to Renilla luciferase signal for each sample. Independent assays were performed from 3–9 times.

### Statistical analysis

To compare rates of mutation between groups chi square or Fisher exact analysis was performed. Luciferase activity of mutant clones was compared using analysis of variance (ANOVA). For all tests p<.05 was considered significant.
